# Supplementary material for: The interactome of intact mitochondria by cross-linking mass spectrometry provides evidence for coexisting respiratory supercomplexes
Source: Mol Cell Proteomics. 2017 Dec 8;17(2):216–32. doi: 10.1074/mcp.RA117.000470 (PMC5795388; doi:10.1074/mcp.RA117.000470)
Supplement: Supplemental Data [file supp_RA117.000470_133922_0_supp_23978_fzffwf.zip › spectra_annotation/mito_DR_spectra_annotation/322-1-14-1-89-1.pdf]

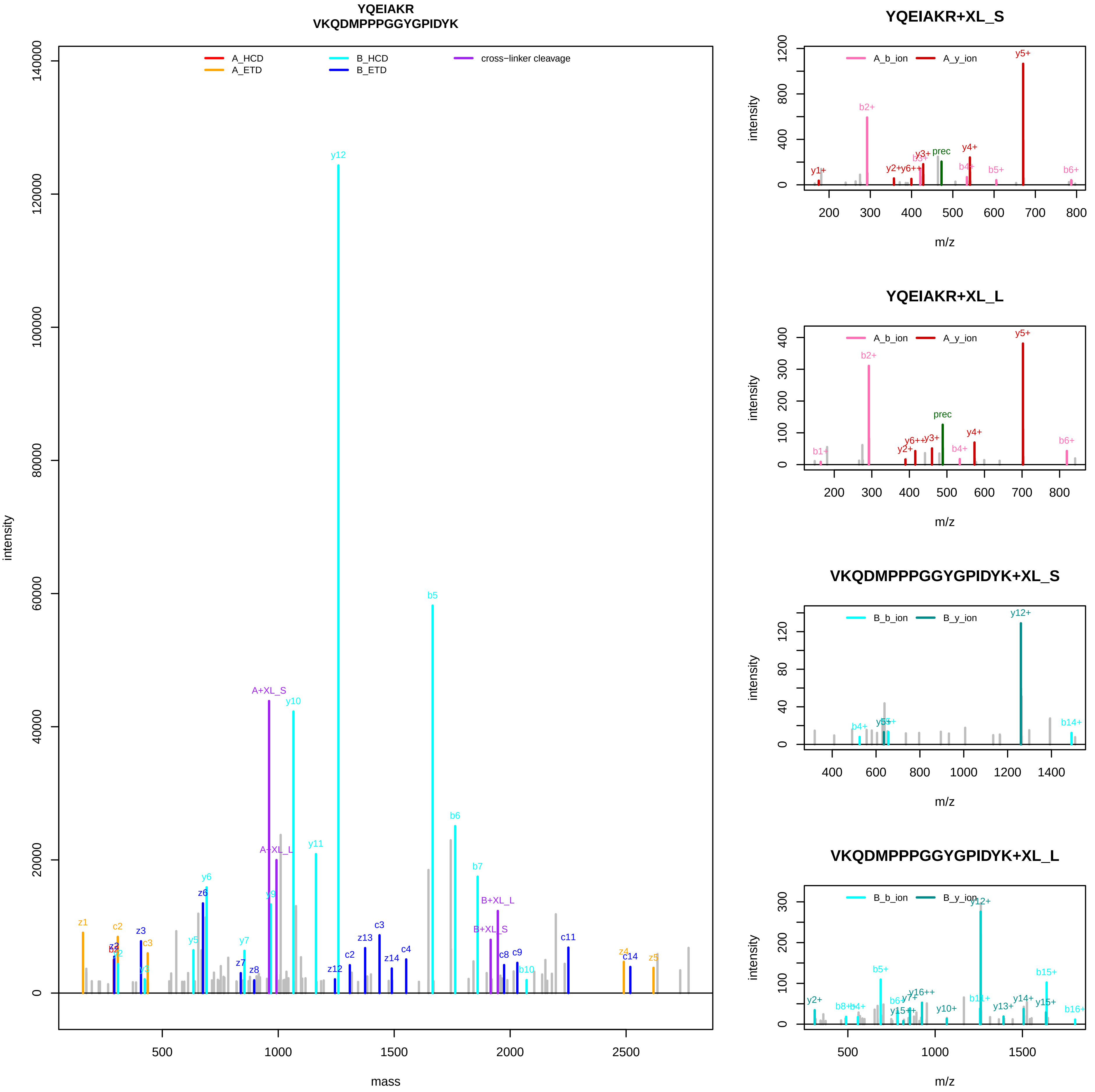
YQEIAKR
VKQDMPPPGGYGPIDYK
A_HCD
A_ETD
B_HCD
B_ETD
cross−linker cleavage
intensity
mass
y12
b5
A+XL_S
y10
A+XL_L
y11
b6
b7
B+XL_L
B+XL_S
y6
z6
y9
z1
c2
z3
c3
y5
y7
z7
z8
c3
z13
c2
z12
z14
c4
c8
c9
b10
c11
z4
c14
z5
YQEIAKR+XL_S
A_b_ion
A_y_ion
y5+
b2+
prec
y4+
y3+
b3+
y2+
y6++
b4+
b5+
b6+
y1+
m/z
YQEIAKR+XL_L
b1+
VKQDMPPPGGYGPIDYK+XL_S
B_b_ion
B_y_ion
y12+
b4+
y5+
b14+
VKQDMPPPGGYGPIDYK+XL_L
b5+
b15+
y2+
b8+
b6+
y7+
y16++
y15++
y10+
y13+
y14+
y15+
b16+
